# Comparison of profile and treatment outcomes between elderly and non-elderly tuberculosis patients in Puducherry and Tamil Nadu, South India

**DOI:** 10.1371/journal.pone.0256773

**Published:** 2021-08-27

**Authors:** Sharan Murali, Yuvaraj Krishnamoorthy, Selby Knudsen, Gautam Roy, Jerrold Ellner, Charles Robert Horsburgh, Natasha Hochberg, Padmini Salgame, Senbagavalli Prakash Babu, Sonali Sarkar

**Affiliations:** 1 Department of Preventive & Social Medicine, JIPMER, Puducherry, India; 2 Section of Infectious Diseases, Boston University School of Medicine, Boston, MA, United States of America; 3 Department of Medicine, Rutgers University, Newark, NJ, United States of America; 4 School of Public Health, Epidemiology & Biostatistics, Boston University, Boston, MA, United States of America; 5 Department of Immunology, Rutgers University, Newark, NJ, United States of America; The University of Georgia, UNITED STATES

## Abstract

The rising geriatric population and the increased susceptibility of this age group to tuberculosis (TB), the deadliest single infectious agent, is bothersome for India. This study tried to explore the demographic and treatment outcome differences between the elderly (aged 60 years and above) and non-elderly TB (<60 years) patients from South India. This study was part of a large ongoing cohort study under the RePORT India consortium. Newly diagnosed TB patients recruited into the cohort between 2014 and 2018 were included in this study. Pretested and standardized questionnaire and tools were used to collect data and were stored securely for the entire cohort. Required demographic, anthropometric and treatment related variables were extracted from this database and analyzed using Stata version 14.0. Prevalence of elderly TB was summarized as percentage with 95% confidence interval (CI). Generalized linear modelling was attempted to find the factors associated with elderly TB. A total of 1,259 eligible TB patients were included into this present study. Mean (SD) of the participants in the elderly and non-elderly group was 65.8 (6.2) and 40.2 (12.0) respectively. Prevalence of elderly TB was 15.6% (95%CI: 13.6%-17.6%) with nearly 71% belonging to 60–69 age category. Male sex, OBC caste, poor education, unemployment, marriage, alcohol consumption and unable to work as per Karnofsky score were found to be significantly associated with an increased prevalence of elderly TB. Unfavorable outcomes (12% vs 6.5%, p value: 0.018), including death (9.3% vs 3.4%, p value: 0.001) were significantly higher among the elderly group when compared to their non-elderly counterparts. The current TB programme should have strategies to maintain follow up with due attention to adverse effects, social support and outcomes. Additional research should focus on predictors for unfavorable outcomes among the elderly TB group and explore ways to handle the same. Rendering adequate social support from the health system side and family side would be a good start.

## Introduction

With nearly 10 million new cases reported in 2019, tuberculosis (TB) is the single infectious agent causing maximum morbidity and mortality globally [1]. India stands prominent in the TB map of the world by contributing to almost one-fourth of the global TB burden [2]. A significant focus of the Global End TB strategy is on reducing the mortality, morbidity, and catastrophic cost due to TB, but progress to the targets has been plodding and is worrisome [1].

The Government of India defines the population over the age of 60 years as elderly [3]. The elderly population in India is on the rise due to the increasing life expectancy and declining fertility rates [4]. The elderly age group is more susceptible to TB disease due to various reasons. Comorbidities, immunosuppressive drugs, malnutrition, chronic alcohol use, and underlying malignant conditions predispose the elderly to TB [5, 6]. The increased notification of TB in older persons (aged 65 years and above) in 2016 globally confirms this [7]. Many countries are now reporting a disproportionate increase in elderly TB compared to the rise in the elderly population [8–10]. Age and nutrition-related factors have also been shown to negatively impact treatment outcomes among elderly TB [11–13]. These could further aggravate the situation by establishing the elderly TB case as a potential source of infection in the community [14–16].

The ever increasing elderly population of India is projected to cross 12.6% of the total population by 2026 [17]. While the overall disease prevalence is high among the 15–30 age group in the country, the South Indian states, including the Union Territory (UT) of Puducherry is showing a higher elderly prevalence. The India TB report has reported this vast disparity in age distribution among the incident cases of TB in the country 2020 [18]. Identifying TB disease in this high-risk population by using an active screening approach is technically and economically not feasible for a low-middle income country (LMIC) like India. But at the same time, failure in identifying and engaging this mounting burden of elderly TB could be a significant challenge to the ongoing efforts by the National TB Elimination Programme (NTEP) in achieving the ambitious end TB targets of the country by 2025 [19].

Evidence-based elderly health-focused treatment protocols and adequate follow-up of elderly TB is the need of the hour for India [20, 21]. A thorough understanding of the epidemiological features and adverse treatment outcomes among elderly TB patients can help address this emerging threat. Although several studies have been conducted worldwide describing the elderly TB population, the evidence on profile and outcomes between elderly and non-elderly TB patients in India is scarce. Hence, we undertook this study to compare the profile and treatment outcomes between elderly and non-elderly TB patients in South India.

## Methods

### Study setting and study population

This study was a part of a large-scale ongoing cohort study under Regional Prospective Observational Research for Tuberculosis (RePORT)-India Consortium [22]. It is conducted by Jawaharlal Institute of Postgraduate Medical Education and Research (JIPMER) in collaboration with Boston University Medical Campus and Rutgers University. It has two prospective observational cohorts: (1) active pulmonary TB cases and (2) their household contacts. Participants with sputum positive for TB were included in this study as Active Pulmonary TB case.

The study encompassed three districts from South India: Puducherry from the UT of Puducherry and two districts from Tamil Nadu adjacent to Puducherry: Villupuram and Cuddalore. Total number of TB cases reported in Puducherry during 2018 was 3489 with only 23 cases reported from the private sector as per the latest report [2]. In Cuddalore and Villupuram, total TB cases reported nearly 3000 cases with 90% reported from public sector alone [2]. Enrollment started in May 2014 in Puducherry, August 2014 in Cuddalore, and November 2015 in Villupuram. The details of the study protocol have been reported hitherto [23–26].

One TB unit (TU) in Puducherry (population ≈ 9.5 million) and two TUs each in Villupuram (population ≈ 3.5 million) and Cuddalore (population ≈ 2.6 million) were selected to recruit study participants. One TU in Puducherry caters to a population of about 950,000 and TUs selected in Villupuram and Cuddalore serve a population of about 500,000 per TU. The TB program is decentralized to the sub-district level. Service delivery in the selected districts at this level is operationalized through the Designated Microscopy Centers (DMCs) and the Peripheral Health Institutions (PHIs) like Primary Health Centers (PHC) and Subcenters with TUs as the nodal point. Diagnosis and treatment for TB are provided free of cost at the TUs. Individuals diagnosed with TB at the DMC are referred to their nearby PHC for initiation of treatment. Screening for comorbidities like Type II Diabetes, HIV, etc., is also done here. Sociodemographic details, their clinical course, medication adherence, and comorbidity profile are maintained at the PHC level and followed up using treatment cards. The same gets updated into the RePORT system. RePORT system does not provide any intervention to TB patients.

All newly diagnosed sputum smear-positive TB patients (at least 1+ acid-fast bacilli [AFB]) more than or equal to six years of age from the selected districts during the study duration 2014 to 2018 were included in this study. We excluded patients with a previous history of TB disease or treatment, drug resistant TB, and patients currently on TB treatment for more than or equal to seven days from the analysis.

### Study procedure

A pretested semi-structured questionnaire was used to capture the sociodemographic characteristics such as age, sex, education (classified as no formal education/primary/secondary/higher), occupation, religion, caste, and marital status. Karnofsky score was used to assess functional impairment [27]. History of current tobacco smoking and alcohol use were self-reported by the participants. Height and weight were measured for calculating the body mass index (BMI).

A majority of the interviews were conducted in the clinic, though some were in the participants’ homes. Filled questionnaires were scanned and transferred to Boston University using the Verity Tele Form Information Capture System software V10.8 (Sunnyvale, CA, USA). It was read into a Microsoft Access (Seattle, WA, USA) database. Errors in the data entry process were reviewed and duly corrected by the on-site team in India. After the baseline assessment, compliance with TB medications and sputum smear examination was done at the end of the intensive phase (2 months after treatment initiation) and the continuation phase (6 months after treatment initiation). At the end of the continuation phase, the treatment outcome was recorded and updated into the same database from the treatment cards.

### Study definitions

#### TB diagnosis

Diagnosis of active TB was made using sputum smear examination—positive for MTB with at least 1+ AFB.

#### Hazardous alcohol user

A participant was classified as a hazardous alcohol user if they have reported drinking alcohol for about 2 to 3 times/week and had ≥3 drinks on a typical day; or 2 to 4 times/month and had ≥6 drinks in one sitting at the least monthly.22 No differentiation was made between males and females.

#### BMI classification

BMI was categorized as underweight (<18.5 kg/m2), normal (18.5–22.9 kg/m2), overweight (23–24.9 kg/m2) and obese (≥25 kg/m2) based on Asian classification [28].

#### Karnofsky score

Patients with score between 80 and 100 were able to carry on normal activity and work without requiring any special care; score between 50 and 70 indicated participants were unable to work but able to live at home and carry out personal needs with varying requirement of assistance; score between 0 and 40 were considered unable to care for self and requires hospital or institutional care [29].

#### Elderly TB

Participants diagnosed with TB and aged ≥60 years were considered as elderly TB patients [3].

#### Compliance to TB medication

Patients who never skipped medications were considered to have good compliance to TB treatment. This data was retrieved from the treatment cards of the patients.

#### TB treatment outcomes [22]

At the end of continuation phase (six months after treatment initiation), outcomes were assessed.

*Bacteriological cure*. Participant completing the drug therapy and has a documented negative culture results at the last month or end of the treatment and at least one previous occasion

*Treatment completed*. Participant who has taken at least 90% of the total doses recommended under the NTEP treatment guidelines

*Favorable treatment outcome*. Participants satisfying the criteria of treatment completed and/or bacteriological cure were classified as having favorable treatment outcome

*Bacteriological failure*. Participant having positive sputum culture at the last month of treatment and the result was not found to be false positive.

*Clinical failure*. Participant who has completed at least four months of treatment regimen, but still have persistent signs and symptoms, progression of disease or recurrence of symptoms and/or signs of TB found to be because of TB and not because of any other underlying causes.

*Emerging resistance*. Participant who had a change in baseline drug sensitivity before the drug sensitive bacteriological failure can be determined (i.e., after baseline visit, but before 5 months of treatment).

*Treatment not completed*. Participants who interrupt or drop out of the treatment regimen for two or more months consecutively, but continues to be in the study follow-up

*Lost to follow-up*. Participants who stopped having their follow-up visits after at least one month of treatment initiation

*Death*. Participant who have died of any cause after enrollment into the study and before the study completion

*Unfavorable treatment outcome*. Participants satisfying any of the following criteria: death, bacteriological/clinical failure, emerging resistance, clinical relapse were classified as having unfavorable treatment outcome

### Statistical analysis

Data were extracted from the RePORT India consortium project database for the JIPMER site and analyzed using Stata v.14.2 software. The demographic and treatment variables for the entire cohort was summarized first. Continuous variables such as age was summarized as mean and standard deviation (SD). Categorical variables were summarized as proportions. Prevalence of elderly TB was summarized as percentage with 95% confidence interval (CI). We used generalized linear model (GLM) applying Poisson regression with robust error variance and a log-link to find the factors associated with elderly TB. We have applied this model to obtain the prevalence ratio (PR), which is more interpretable and easy to communicate with the clinicians than odds ratio (OR) [30]. In addition, OR tends to inflate the association if the prevelance of outcome is more than 5%. Hence, Poisson regression model with robust error variance is a suitable alternative to the traditional logistic regression analysis [30]. Sex, education, occupation, marital status, religion, caste, Karnofsky score, BMI, comorbidity, smoking and alcohol use were considered as covariates. Factors with p value less than 0.2 in the univariable analysis were considered for multivariable analysis using forward model building. Unadjusted and adjusted PR were obtained from univariable and multivariable analysis. Variables with p value less than 0.05 in the final model were considered as statistically significant. Difference in compliance to medications and TB treatment outcomes at the end of follow-up between elderly and non-elderly TB patients was assessed using Chi-square test and p value less than 0.05 was considered statistically significant.

### Ethical considerations

Informed written consent or assent in addition to parents’ consent (in case of participant <18 years) was obtained from all participants enrolled in the study. The study protocol was approved by the Institute Ethics Committee and Scientific Advisory Committee of JIPMER, and the Institutional Review Boards at Boston University Medical Campus and Rutgers-New Jersey Medical School.

## Results

### Sociodemographic, behavioral and anthropometric characteristics

A total of 1,259 eligible TB patients were included during the study period. Sociodemographic, behavioral and anthropometric characteristics of the study participants are described in Table 1. The mean (SD) age of the study participants was 43.7 (14.6) years, with mean (SD) in non-elderly group was 40.2 (12) years and in elderly group was 65.8 (6.2) years. Majority (985; 78.2%) of the participants were males and 944 (75.2%) were employed; about 204 (16%) participants did not have any formal education; majority (1119 participants; 89.2%) were Hindus; about 909 participants (72.3%) were currently married; about 335 participants (27%) belonged to Scheduled Caste (SC) /Scheduled Tribe (ST) category. Majority (765 participants; 61.2%) were underweight; about 741 participants (58.9%) were alcohol users and 311 participants (24%) were smokers. Performance status assessed by Karnofsky score showed that only 364 participants (29%) were able to carry on normal activity and to work without the requirement of special care.

**Table 1:** Profile of the study participants across the 2 groups—elderly & non-elderly (N = 1259).

	Characteristic	Total	Elderly TB	Unadjusted PR (95% CI)	P value	Adjusted PR (95% CI)	P value
		**n (%)**	**n (%)**				
1	**Sex**						
	Male	985 (78.2)	164 (83.2)	1.4 (0.95–2.01)	0.06	3.4 (2.07–5.61)	**<0.001**
	Female	274 (21.8)	33 (16.8)	(ref)	-	(ref)	**-**
2	**BMI Category** * **(N = 1250)**						
	Underweight (<18.50)	765 (61.2)	109 (55.3)	0.8 (0.57–1.05)	0.07	0.8 (0.57–1.17)	0.28
	Normal (18.50–22.99)	365 (29.2)	67 (34.0)	(ref)	-	(ref)	-
	Overweight (23.00–24.99)	65 (5.2)	8 (4.1)	0.7 (0.32–1.40)	0.24	0.7 (0.33–1.44)	0.32
	Obesity (≥ 25.00)	55 (4.4)	13 (6.6)	1.3 (0.71–2.33)	0.35	1.0 (0.55–1.88)	0.95
3	**Religion (N = 1255)**						
	Hindu	1119 (89.2)	178 (0.8)	(ref)	-	*Not included in model*	
	Christian	82 (6.5)	9 (4.6)	0.7 (0.35–1.35)	0.24		
	Islam	54 (4.3)	9 (4.6)	1.0 (0.54–2.05)	0.88		
4	**Caste (N = 1239)**						
	SC/ST	335 (27.0)	39 (20.2)	(ref)	-	(ref)	
	OBC	904 (73.0)	154 (79.8)	1.5 (1.02–2.07)	**0.03**	1.6 (1.08–2.32)	**0.01**
5	**Educational Status (N = 1254)**						
	No formal education	204 (16.3)	59 (30.1)	7.6 (3.79–15.42)	**<0.001**	5.2 (2.46–11.02)	**<0.001**
	Primary	287 (22.9)	59 (30.1)	5.4 (2.69–10.96)	**<0.001**	3.7 (1.78–7.71)	**<0.001**
	Secondary	525 (41.9)	69 (35.2)	3.4 (1.73–6.96)	**<0.001**	2.5 (1.21–5.12)	**0.01**
	Higher	238 (19.0)	9 (4.6)	(ref)	-	(ref)	**-**
6	**Employment status (N = 1258)**						
	Unemployed	114 (9.1)	47 (23.9)	3.0 (2.16–4.21)	**<0.001**	2.4 (1.69–3.54)	**<0.001**
	Employed	944 (75.2)	129 (65.5)	(ref)	-	(ref)	
	Others	198 (15.7)	21 (10.7)	0.8 (0.48–1.23)	0.25	1.3 (0.77–2.18)	0.33
7	**Marital status (N = 1257)**						
	Currently married	909 (72.3)	147 (74.6)	5.0 (2.37–10.80)	**<0.001**	3.2 (1.34–7.57)	**0.008**
	Never married	219 (17.4)	7 (3.6)	(ref)	-	(ref)	
	Widowed/Divorced/Separated	129 (10.3)	43 (21.8)	10.4 (4.69–23.18)	**<0.001**	6.9 (2.78–17.3)	**<0.001**
**8**	**Comorbidity**						
**A**	**Diabetes (N = 1186)**						
	Absent	791 (66.7)	112 (59.3)	(ref)	-		
	Present	395 (33.3)	77 (40.7)	1.4 (1.03–1.83)	**0.03**	1.2 (0.83–1.68)	0.35
**B**	**HIV (N = 1259)**						
	Absent	1056 (99.4)	197 (100.0)	*Not included in model*			
	Present	6 (0.5)	0 (0)				
**C**	**Others** ** **(N = 1259)**						
	Absent	1226 (97.4)	195 (99.0)	(ref)	-		
	Present	33 (2.6)	2 (1.0)	0.4 (0.09–1.53)	0.19	0.6 (0.16–2.67)	0.56
**9**	**Habits (N = 1257)**						
**A**	Smoker^#^	311 (24.7)	50 (25.4)	1.0 (0.75–1.42)	0.82	*Not included in model*	
**B**	Alcohol user^#^	741 (58.9)	99 (50.2)	0.7 (0.53–0.93)	**0.01**	0.4 (0.29–0.58)	**<0.001**
**10**	**Karnofsky** ^#^ **(N = 1257)**						
	Able to carry normal activity and work	364 (29.0)	47 (23.9)	(ref)	-	(ref)	
	Unable to work, able to live at home	893 (71.0)	150 (76.1)	1.3 (0.94–1.82)	0.09	1.4 (1.01–2.02)	**0.04**

(1) Total and elderly TB are summarized as frequency with proportions.

(2) Keeping elderly TB as outcome, unadjusted (Unadjusted PR) & adjusted prevalence (Adjusted PR) ratios with its Confidence Intervals (95% CI) was calculated using MEGLM modelling using a poisson family and log link. (N = 1259).

(3) Religion and Current tobacco use were not included into the model as the p value was more than 0.20

# Self-reported by the participants |

*Asia Pacific guidelines for obesity

**Other Co- morbidity include: Asthma (2), Urinary Calculus (11) & Hepatitis (20)

### Comparison of sociodemographic, behavioral and anthropometric profile between elderly and non-elderly TB patients

Of the total participants, 197 (15.6%; 95%CI: 13.6%-17.6%) had elderly TB. Out of these 197 elderly TB patients, 152 (77%) belonged to the 60–69 age category. Among the rest, 41 (21%) belonged to 70–79 and 4 participants (2%) were aged 80 years and above. Table 1 documents the comparison of sociodemographic, behavioral and anthropometric profile between elderly and non-elderly TB patients. In unadjusted analysis, there was significant difference between elderly and non-elderly TB patients in terms of education (p<0.001), marital status (p<0.001), occupation (p<0.001), caste (p = 0.03), presence of diabetes mellitus as comorbidity (p = 0.03), and alcohol use (p = 0.01). Apart from these variables, sex, BMI category, Karnofsky score, presence of other comorbidities in the patient were also included in the final multivariable model as these variables had p value less than 0.2.

The adjusted analysis was performed with the above-mentioned variables and the final model had 1,157 participants owing to missing data with respect to one or more of the covariates. Male sex was significantly associated with elderly TB (aPR = 3.4; 95% CI: 2.07–5.61; p<0.001), after adjusting for potential confounders. Patients with no formal education had 5.2 times higher prevalence of being in elderly TB group compared to those having higher educational qualification (aPR = 5.2; 95%CI: 2.46–11.02; p<0.001) and this association was statistically significant. Unemployed TB patients had 2.4 times more chance of being in the elderly TB group compared to those employed TB patients (aPR = 2.4; 95%CI: 1.69–3.54; p<0.001). OBC caste was also significantly associated with elderly TB (aPR = 1.6; 95%CI: 1.08–2.32; p = 0.01). Patients who are unable to work with varying assistance needed were 1.40 times more likely to be in elderly TB group compared to those who were able to work without any special care required (aPR = 1.40; 95%CI: 1.01–2.02; p = 0.04). Alcohol drinkers were significantly associated with non-elderly TB (aPR = 0.40; 95%CI: 0.29–0.58; p<0.001) (Table 1).

### Comparison of compliance and outcomes between elderly and non-elderly TB patients

**Fig 1:**
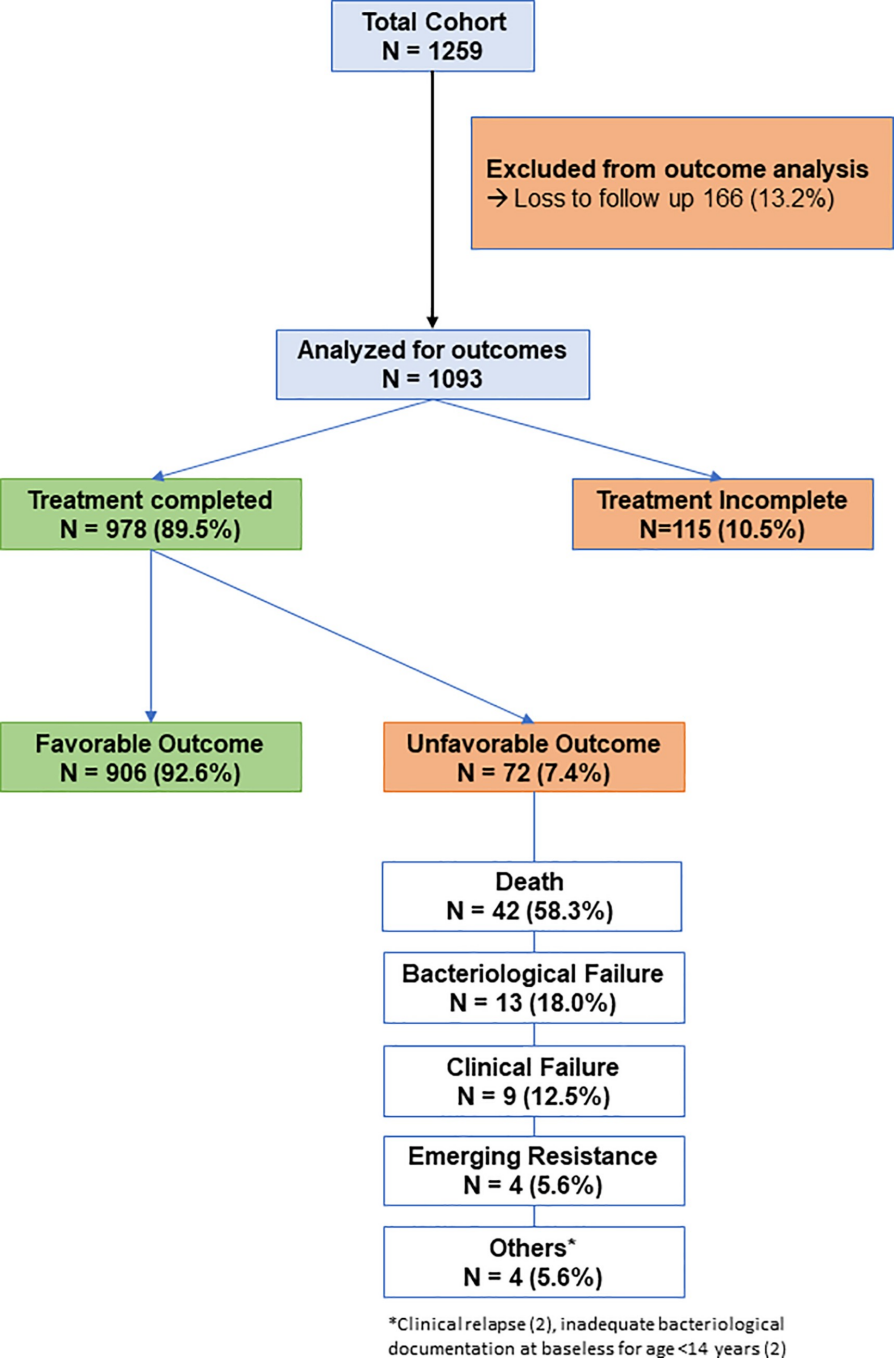
Summary of flow of participants into various stages during the follow up of the study.

Fig 1 explains the flow of participants through the study.

**Fig 2:**
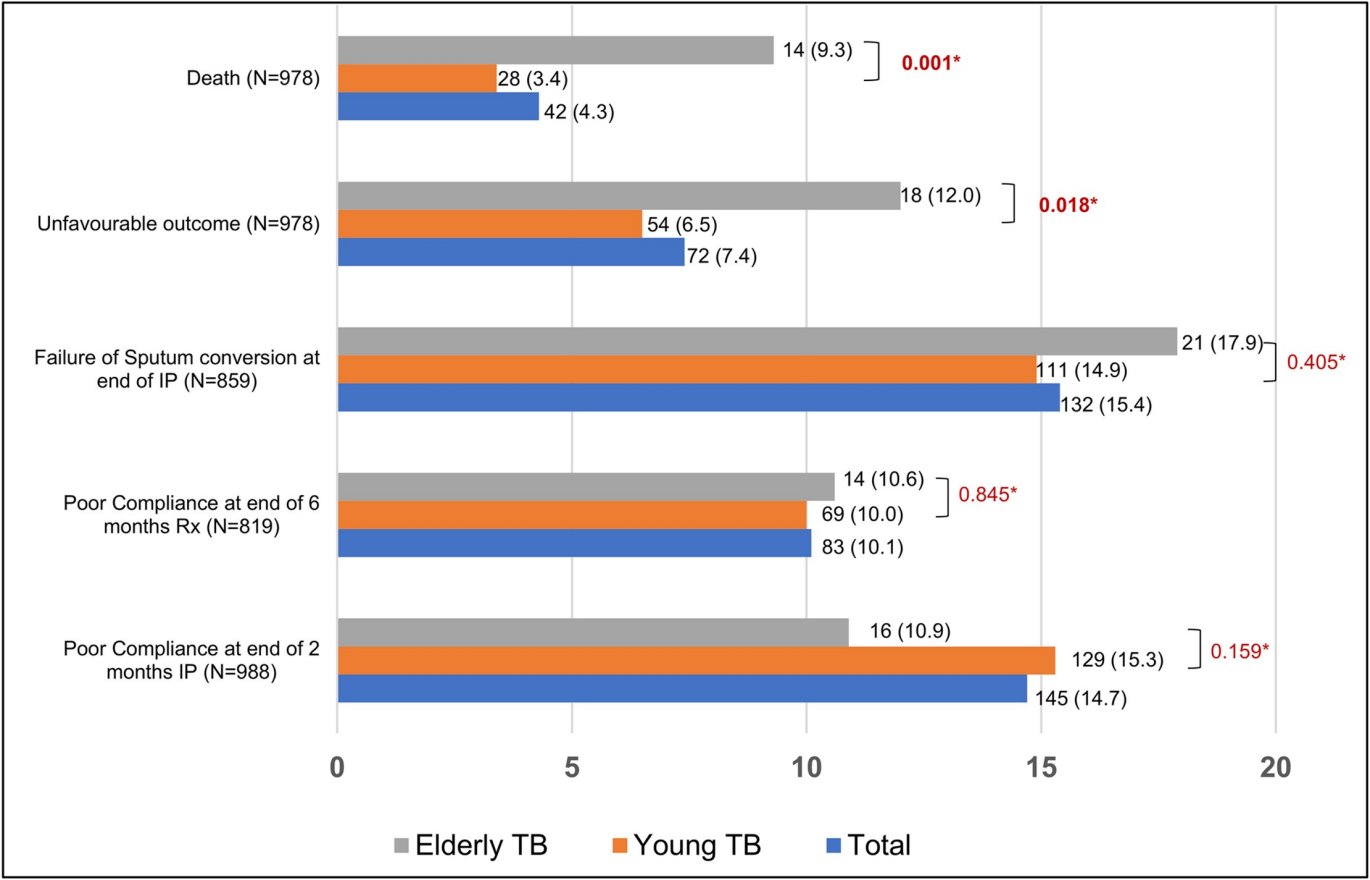
Comparing selected poor outcomes across the two study groups, elderly and non-elderly TB. (1) The denominator for each outcome is written in parenthesis with the axis title. (2) Frequency (proportion) of the outcome in the study group–non-elderly or elderly TB is given as value labels. *p values of comparison of outcome between the two groups, obtained by Chi-Square test.

Compliance data was available for only 988 patients at the end of intensive phase and 819 patients at the end of continuation phase. From the treatment initiation till the end of intensive phase, 145 out of 988 patients (14.7%) had poor compliance to medications, and 83 out of 819 patients (10.1%) had poor compliance between End of intensive and End of continuation phase. Overall, during the six months follow-up period, 209 out of 819 patients (25.5%; 95%CI: 22.5%-28.5%) had poor compliance to medications. Poor compliance was higher among non-elderly TB patients compared to elderly TB patients (26.3% vs 21.1%). However, this difference was not statistically significant (p = 0.21). Sputum conversion at the end of two months was achieved among 84.6% of the study participants. Sputum conversion was found to be better among the non-elderly TB patients (85.0%) compared to elderly TB patients (82.0%), but this difference was not statistically significant (p = 0.40) (Fig 2).

From among the 1,259 participants in the original cohort, at the end of 6 months, 166 (13.2%) participants were lost to follow up with almost equal proportion in both elderly (14.7%) and non-elderly group (12.9%) (p value: 0.488). About 115 (9.4%) did not fully complete their treatment with almost equal proportion, i.e., 9.4% of elderly and the same of non-elderly TB maintained an incomplete treatment status (p value: 0.988).

Forty-two (4.3%) participants were reported dead at the end of 6 months follow up. A higher proportion of elderly TB patients (9.3%) were marked dead than the non-elderly participants (3.4%) and this difference was found to be statistically significant (p value = 0.001). Treatment failure (bacteriological or clinical) was found among 2.2% of study participants with outcome data available. Overall prevalence of unfavorable outcomes among the study participants was 7.4% (95%CI: 5.7%-9.0%). Unfavorable outcomes were more common among elderly TB patients (12%) compared to non-elderly TB patients (6.5%), and this difference was found to be statistically significant (p = 0.018) (Fig 2).

## Discussion

TB in the elderly population has emerged as an important public health issue globally. The outcome in this group is crucial in the control of disease and success of the National TB Elimination Program [31]. The higher proportion of elderly TB in the present cohort (15%) could be explained by the higher elderly population in Tamil Nadu (10%) and Pondicherry (10%) as per the India Census 2011 data [32]. Elderly TB patients were more likely to be males, have no formal education, unemployed, requiring assistance and less alcohol users compared to the non-elderly TB patients, the difference being statistically significant. Compliance to medications was better among the elderly TB patients, while the treatment outcomes at the end of intensive and continuation phase were better among non-elderly TB patients and this difference was statistically significant.

The proportion of elderly TB observed in our study (14.9%) is similar to that observed in previous studies conducted in India such as Karnataka (18.7%) [33], Tamil Nadu (14%) [34] and Punjab (12.3%) [35] and other countries such as Nigeria [36], Uganda [8], Taiwan [9] and United States [10]. However, this might be expected to increase over time given the increasing life expectancy in India. The existing TB control program is silent about any specific policies/programs/schemes for elderly patients with TB. This policy or program gaps is of concern given expected rising problem of TB in the elderly. Hence, this group should be given special attention and have targeted intervention to improve the success of the TB control program.

We found that elderly males have higher rate of TB compared to females in South India. This finding is in line with the epidemiological trend of TB in India (males are more affected than females) [37, 38]. Previous studies conducted in India and other LMIC countries have also reported that elderly males have higher rate of TB than females [33–36]. Possible reasons for this pattern could be better healthcare service accessibility and higher rate of social interaction among males [39, 40]. We also found that elderly patients were more likely to be unemployed than non-elderly TB. This might be attributable to poor health seeking behavior among these individuals, as the costs involved in seeking health care might be unaffordable for the unemployed individuals, leading to delayed diagnosis of the condition [41]. We also identified OBC caste as a significant determinant of TB among elderly patients, which indicates the social and economic differences existing across the region. This might limit the treatment seeking behavior leading to delayed diagnosis as TB among elderly patients. Regular screening for active case finding of TB, increased awareness about the importance of early testing and mobile clinics could help in early case identification. This can be substantiated by the better adherence despite their lower Karnofsky scores.

Sputum smear conversion at the end of intensive phase is an early indicator for successful TB treatment outcome [42]. Though compliance to medication was better among the elderly patients, sputum conversion at end of intensive phase was lower among elderly patients compared to non-elderly patients. Though not statistically significant, a similar finding was reported in previous studies conducted in India where the sputum conversion was lower among the TB patients in elderly age group [33–35]. Possible reasons for this lower conversion rate could be due to the high bacillary load among the elderly patients leading to delayed clearance and sputum conversion, reported in some studies [43]. Another reason could be the higher rate of adverse reactions, drug interactions, and comorbidities leading to dosage reduction and poor absorption of anti-tubercular drugs among the elderly [44].

The rate of unsuccessful treatment outcomes such as bacteriological or clinical failure, death were more among elderly compared to non-elderly TB patients. However, certain undesirable outcomes such as lost to follow-up and treatment incomplete status, though the most commonly found among the total study participants, it was not significantly different between elderly and non-elderly group. This finding was in line with the previous studies conducted around the world (high- and low-income countries alike like Hong Kong, Taiwan, Uganda, Nigeria, United States) and also past evidences in India [8–10, 33–35, 45–50]. Reason for such unsuccessful outcomes could be the higher rate of comorbidities and advanced/severe form of disease among elderly [51, 52]. Hence, elderly patients should be provided extra care and attention to reduce the extent of unsuccessful outcomes. Target groups among elderly patients at risk of unsuccessful outcomes can be identified and close monitoring of such patients can be done. However, there is still a major policy gap exist in the current TB control program. Future TB surveys should have a specific focus on the elderly age group, while the national TB programs should consider performing active case finding among the elderly population and include them in their preventive strategies.

In India, elderly population provides the careers for the societies, leaders and mentors for the communities, and also play a major role in educating and motivating the young generation throughout the country [53]. However, it is common for the elderly to be stressed by various factors like the loss of spouse, high income insecurity and compulsion to work, poor nutrition, rising incidence of non-communicable diseases including mental illnesses [32]. High incidence of poor outcomes among the elderly TB patients complicates this situation further [54]. Support from the family or social support systems during these crucial times are necessary for improving the general quality of life. Understanding and quantifying the level of support necessitated by the elderly TB patients would be a novel addition to the model and can be taken up in future studies. However, caring for the rising numbers of elderly with TB might prove to be difficult in the resource constrained regions. Still, the care and attention for elderly TB should be improved as much as the attention provided currently to the burden of childhood TB patients (≤14 years). Health systems must confront these challenges of the aging population and provide integrated services required for addressing the health needs of the elderly. Treatment regimens should also be framed specific to the elderly population just like the childhood TB patients. For this purpose, further research should emphasize on the inclusion of elderly in the development of newer TB drugs and assessing the effectiveness of existing drugs and regimens [53]. Our study has several strengths. Firstly, our study adds to the limited evidence available on comparison of sociodemographic, behavioral, anthropometric profile, compliance and treatment outcomes between non-elderly and elderly TB patients. Longitudinal analysis of compliance and treatment outcomes data will help in causal inference between the exposure and outcomes. We also have comprehensively covered majority of the newly diagnosed TB patients receiving care at DOTS centers during the study period. High sample size, response rate, quality assurance of data through double data entry & validation are added advantages of the study. We also used validated questionnaires such as AUDIT-C, Karnofsky score for the assessment of behavioral and mobility profile of patients. All these factors together enable valuable comparisons with other TB cohorts across India and other similar settings.

Still, our study has certain limitations. TB patients included in our study were from three selected districts in South India which might limit the generalizability of the study findings. In addition, only the patients diagnosed in public healthcare facilities were included. Hence, the sample may not be representative of patients diagnosed in private health sector. In spite of these limitations, our study has several programmatic implications. First, we provided baseline comparison of profile between non-elderly and elderly people with large-scale representation of TB patients. We also identified specific target groups–male sex, underweight, OBC, non-formal education, unemployment, widowed, divorced individuals etc—requiring additional attention and focused interventions. Policy makers and program managers can make use of this information to develop appropriate interventions aimed at increasing successful management of TB among this vulnerable population.

## Conclusion

With a prevalence of 16%, TB among elderly patients stands out as an urgent and important concern that needs to be handled for effective control of the ongoing TB pandemic. Non-elderly TB patients were poorly compliant than the older ones, but the outcomes were statistically unfavorable in the latter. TB among elderly patients causes more deaths and other unfavourable outcomes. The need for more focused attention to this group of TB patients is more relevant considering our strive to achieve the ambitious targets of End TB in India by 2025.

## Supporting information

S1 File Data collection form. (PDF)

S1 Dataset Elderly TB–full dataset. (DTA)
