# Supplementary material for: Comparison of profile and treatment outcomes between elderly and non-elderly tuberculosis patients in Puducherry and Tamil Nadu, South India
Source: PLoS One. 2021 Aug 27;16(8):e0256773. doi: 10.1371/journal.pone.0256773 (PMC8396735; doi:10.1371/journal.pone.0256773)
Supplement: S1 File — (PDF) [file pone.0256773.s001.pdf]

Indo-US VAP Biomarkers for Risk of Tuberculosis and for Tuberculosis Treatment Failure and Relapse

Form 2A

INDEX CASE: Clinical/Demographic Form

This questionnaire must be completed for each Index Case as the baseline visit. The purpose of this questionnaire is to evaluate the clinical characteristics and infectiousness of the Index TB case.

Index Case ID Number: 1 0 2 - 0 _ _ _ _ - A

PHC ID *(Refer to code sheet):* ☐☐☐

Date of evaluation: ☐☐ / ☐☐ / ☐☐☐☐
Day Month Year

CLINIC IDENTIFICATION ☐ Not Applicable
Indicate the participant's DMC or TU if known:

Designated Microscopy Center (DMC): ☐☐☐

Tuberculosis Unit (TU): ☐☐

We would like to ask you some questions about your health, including information on living conditions and health facilities. This survey will take about 15 minutes. The information you provide will be used to better understand how tuberculosis affects people. Your answers will remain anonymous. Your name will not be written on the questionnaire. At any time, if there is a question that you'd rather not answer, please let me know and we can skip that question. Some of the questions may be sensitive, but please do your best to answer honestly. At this time do you want to ask me anything about the survey? *(Answer any questions and address respondent's concerns.)*

May I start the interview now? *(Proceed if participant agrees.)*

We will begin with a few questions about your background. *(If the participant is a child or otherwise unable to respond and the guardian is not present, say, "*You may want your parent/guardian to come help you answer questions.*").*

DEMOGRAPHIC INFORMATION

1. Are you married?

- ☐ Never married
- ☐ Married/Living together
- ☐ Separate/divorced
- ☐ Widowed
- ☐ Not applicable (child)

2. What is your religion?

- ☐ Christian
- ☐ Hindu
- ☐ Muslim
- ☐ Sikh
- ☐ Jain
- ☐ Buddhist
- ☐ Zoroastrian (Parsi)
- ☐ Not religious
- ☐ Other, specify: ____________
- ☐ Refused to answer
- ☐ Don't know

3. What is the caste or tribe of the head of the household?

- ☐ Scheduled caste
- ☐ Scheduled tribe
- ☐ Other backward caste
- ☐ None of them
- ☐ No caste or tribe
- ☐ Don't know

4. What is your mother tongue?

- ☐ Assamese
- ☐ Bengali
- ☐ English
- ☐ Gujrathi
- ☐ Hindi
- ☐ Kannada
- ☐ Kashmiri
- ☐ Konkani
- ☐ Malyalam
- ☐ Manipuri
- ☐ Marathi
- ☐ Nepali
- ☐ Oria
- ☐ Punjabi
- ☐ Sindhi
- ☐ Tamil
- ☐ Telgu
- ☐ Urdu
- ☐ Other, specify: ____________

5. For how many years did your mother go to school?

☐☐ years *(Enter 00 if mother did not go to school.)*

☐ Refused to answer

☐ Don't know

6. For how many years did you go to school?
(Must be formal education with classes and grades)

☐☐ years *(Enter 00 if subject did not go to school.)*

☐ Refused to answer

☐ Don't know

7. Before you became ill, were you working, unemployed, a housewife or a student?

☐ Employed

☐ Student

☐ Housewife

☐ Unemployed

☐ Other, specify: ____________________

8. Have you ever worked in mining, road or building construction, stone cutting, or glass manufacturing?

☐ Yes (Go to 8a) ☐ No

8a. If YES, for how many years did you do this work?

☐☐ years

9. Before you were sick, what was your entire household income in a month?
If participant is unclear, help them add together the incomes of all household members.

☐ < Rs 3000

☐ Rs 3000-5000

☐ Rs 5001-10000

☐ > Rs 10000

☐ Refused to answer

☐ Don't know

10. How many wage earners are there in this house?

☐☐ wage earners

11. What color is your ration card?

☐ Red ☐ Yellow ☐ Green ☐ Other

(Refer to PHC Population List)

12. The home location is:

☐ Large city (over 10 lakh people)

☐ Small city (50,000 to 10 lakh people)

☐ Town (other urban area) ☐ Rural (countryside)

TUBERCULOSIS SOURCE EVALUATION

I am going to ask you questions about your household. By household, I mean others who eat from the same pot.

13. How long have you been living in your current home?

☐☐ years **OR** ☐☐ months

14. Over the last 3 months, how many hours per day do you spend at your home on average?
If this can't be recalled, go through the day with them.

☐ Less than 6 hours per day

☐ Between 6-12 hours per day

☐ Between 13-18 hours per day

☐ Over 18 hours per day

☐ Don't know

15. Has anyone that you've lived with in the past 2 years been diagnosed with tuberculosis?

☐ Yes (Go to 14a)

☐ No

☐ Don't Know

14a. If YES, was this person told he/she had tuberculosis before you or after you were diagnosed with tuberculosis?

☐ Before

☐ After

☐ Don't Know

10334

Index Case ID Number: 1 0 2 - 0 _ _ _ _ - A

TUBERCULOSIS KNOWLEDGE

The next questions ask about your knowledge and opinions on tuberculosis disease

16. How does tuberculosis spread from one person to another?
(Multiple Responses Possible. Read options aloud)

- ☐ Through the air when coughing or sneezing
- ☐ Through sharing utensils
- ☐ Through sharing clothes/bed sheets/towels
- ☐ Through smoking bidis/cigarettes/tobacco
- ☐ Through touching a person with tuberculosis
- ☐ Through food
- ☐ Through sexual contact
- ☐ Through mosquito bites
- ☐ Don't know
- ☐ Other

17. Can tuberculosis be cured?

- ☐ Yes
- ☐ No
- ☐ Don't know

RISK BEHAVIOURS

Now I am going to ask you some questions about your use of alcoholic beverages during this past year.

Use the alcohol Beverage Card to show the different types and sizes of drinks.

18a. How often do you have a drink containing alcohol?

- ☐ Never (skip to question 18)
- ☐ Monthly or less
- ☐ 2 to 4 times a month
- ☐ 2 to 3 times a week
- ☐ 4 or more times a week

18b. How many drinks containing alcohol do you have on a typical day when you are drinking?
(Use the Beverage Card to show the different types and sizes of drinks)

- ☐ 1 or 2
- ☐ 3 or 4
- ☐ 5 or 6
- ☐ 7, 8 or 9
- ☐ 10 or more

18c. How often do you have six or more drinks on one occasion?

- ☐ Never
- ☐ Less than monthly
- ☐ Monthly
- ☐ Weekly
- ☐ Daily or almost daily

Now I will ask you a few questions about your smoking habits.

19. Have you ever smoked or used tobacco?

- ☐ Yes, current smoker / user (Go to 19a)
- ☐ Yes, former smoker / user (quit at least 1 month ago) (Go to 19a)
- ☐ No (Skip to 20)

19 a. Which of the following have you ever smoked regularly:

☐ Beedis, hand-rolled cigarettes, or manufactured unfiltered cigarettes → *If checked ask the following questions:*

How long have you smoked these? ☐☐ years OR ☐☐ months

Over the time that you've smoked, around how many did you smoke per day? ☐☐

On average, how many do you currently smoke per day? ☐☐
Enter 00 if not a current smoker

If you are not currently smoking these days, how long has it been since you quit? ☐☐ years OR ☐☐ months

☐ Manufactured filtered cigarettes → *If checked ask the following questions:*

How long have you smoked these? ☐☐ years OR ☐☐ months

Over the time that you've smoked, around how many did you smoke per day? ☐☐

On average, how many do you currently smoke per day? ☐☐
Enter 00 if not a current smoker

If you are not currently smoking these days, how long has it been since you quit? ☐☐ years OR ☐☐ months

☐ Cigars, cheeroots, or cigarillos → *If checked ask the following questions:*

How long have you smoked these? ☐☐ years OR ☐☐ months

Over the time that you've smoked, around how many did you smoke per day? ☐☐

On average, how many do you currently smoke per day? ☐☐
Enter 00 if not a current smoker

If you are not currently smoking these days, how long has it been since you quit? ☐☐ years OR ☐☐ months

☐ Hookah → *If checked ask the following questions:*

How long have you smoked these? ☐☐ years OR ☐☐ months

When you smoked these, around how long did you smoke per day? ☐☐ hours OR ☐☐ minutes

On average, how much time do you currently spend smoking per day? ☐☐ hours OR ☐☐ minutes
Enter 00 if not a current smoker

If you are not currently smoking these days, how long has it been since you quit? ☐☐ years OR ☐☐ months

☐ Other, specify: ____________________ → *If checked ask the following questions:*

How long have you smoked these? ☐☐ years OR ☐☐ months

Over the time that you've smoked, around how many did you smoke per day? ☐☐

On average, how many do you currently smoke per day? ☐☐
Enter 00 if not a current smoker

If you are not currently smoking these days, how long has it been since you quit? ☐☐ years OR ☐☐ months

19 b. Which of the following tobacco products do you use regularly:

☐ Chewing Tobacco → *If checked ask the following questions:*

How long have you used this? ☐☐ years OR ☐☐ months

Over the time that , around how many did you use this per day? ☐☐

On average, how many do you currently use per day? ☐☐
Enter 00 if not a current smoker

If you are not currently using this these days, how long has it been since you quit? ☐☐ years OR ☐☐ months

☐ Snuff → *If checked ask the following questions:*

How long have you used this? ☐☐ years OR ☐☐ months

Over the time that , around how many did you use this per day? ☐☐

On average, how many do you currently use per day? ☐☐
Enter 00 if not a current smoker

If you are not currently using this these days, how long has it been since you quit? ☐☐ years OR ☐☐ months

CLINICAL EVALUATION

I will now ask you some questions about your past health and how you are feeling. *(If the participant is a child, the parent or guardian may respond.)*

20. Have you ever had any health problems other than TB?

☐ Yes (Go to 20a) ☐ No (Skip to 21)

20a. Have you ever been diagnosed with or been told you have the following:

(Read aloud)

☐ Cancer

☐ Partial or complete removal of your stomach

☐ Hepatitis (infection of the liver) or yellowing of the eyes or skin

☐ Chronic kidney failure requiring hemodialysis

☐ Shortness of breath, wheezing or coughing at least three months of the year for two years in a row

ASTHMA

21. Asthma is a chronic disease with repeated attacks of breathlessness or wheezing. Have you been diagnosed with asthma by a doctor?

☐ Yes (Go to 21a) →

☐ No

☐ Don't know

21a. Are you currently using any inhalers? Inhalers are medications that you breathe in to help with asthma.

☐ Yes (Go to 21b and c) →

☐ No

21b. How many inhaler medications do you use in a normal day?

☐ 1 ☐ 3

☐ 2 ☐ >3

21c. Do you use inhalers daily or only when you have trouble breathing?

☐ Daily

☐ With symptoms

HIV

22. Have you been tested for HIV in the past 6 months?

☐ Yes (Go to 22a)

☐ No ☐ Don't know

22a. Where were you last tested?

☐ GCC ☐ DTC ☐ Cuddalore

☐ IGMCRI ☐ JIPMER ☐ Villupuram

☐ Other, specify: ____________________

DIABETES MELLITUS

23. Have you ever been diagnosed with Diabetes Mellitus?

☐ Yes (Go to 23a-b)

☐ No

☐ Don't know

23a. In what year were you diagnosed? ☐☐☐☐

23b. How have you managed your diabetes this past month?

☐ Insulin injections

☐ Oral medication

☐ Other injectable medications

☐ Dietary changes only

☐ Unknown

☐ Other (specify): ____________________

24. When you first became sick this time, where did you first seek healthcare?

☐ Private allopathic clinic ☐ PHC ☐ Medicine shop/pharmacy

☐ Municipal corporation hospital ☐ Medical college hospital ☐ Other, specify: ____________________

☐ Non-allopathic clinic ☐ Government hospital

25. Have you had any of the following signs/symptoms within the past 4 weeks?

Cough ☐ Yes ☐ No

Duration: ☐☐ weeks ☐ Unknown

If duration unknown, ask:
Has your cough lasted:

☐ 0-14 days

☐ 15-28 days

☐ > 28 days

Coughing up blood? ☐ Yes ☐ No

Fever ☐ Yes ☐ No ☐ Unknown

Duration: ☐☐ weeks ☐ Unknown

Night sweats ☐ Yes ☐ No ☐ Unknown

Duration: ☐☐ weeks ☐ Unknown

Unexpected weight loss ☐ Yes ☐ No ☐ Unknown

Duration: ☐☐ months ☐ Unknown

Weight loss: ☐☐ kg ☐ Unknown

**Loss of appetite if adult or failure to gain weight if a child**	☐ Yes ☐ No ☐ Unknown
**Pain when taking a deep breath or coughing that you feel in your chest**	☐ Yes ☐ No ☐ Unknown
**Fatigue**	☐ Yes ☐ No ☐ Unknown
**Any other symptoms, if yes, specify:** ____________________	☐ No ☐ Unknown

26. Karnofsky Score:

☐☐☐ % *Please refer to card for Karnofsky scale.*

27. Do you have any prescriptions from the doctor or pill bottles you are taking medication from?

Ask to see the participant's perscriptions and/or pill bottles if they have any. Check the names of any medications you see on the list below:

☐ None ☐ Decdak ☐ Fluricot ☐ Solumendrol

☐ Betnelam ☐ Decmax ☐ Hisone ☐ Wysolone

☐ Betnesol ☐ Dexona ☐ Omnocortil

DIETARY QUESTIONS

Now I will ask you some questions about the food people in your household eat. We recognize that sometimes it can be hard to get food, so we want to understand this a little more. *(If the participant is a child or otherwise unable to respond, the parent, guardian or other household member may respond.)*

Rarely = once or twice in the past 4 weeks

Sometimes = 3-10 times in the past 4 weeks

Often = more than 10 times in the past 4 weeks

28a. In the past 4 weeks how often was there ever no food to eat of any kind in your household because of lack of resources to get food?

☐ Never ☐ Rarely ☐ Sometimes ☐ Often

28b. In the past 4 weeks how often did you or any household member go to sleep at night hungry because there was not enough food?

☐ Never ☐ Rarely ☐ Sometimes ☐ Often

28c. In the past 4 weeks how often did you or any household member go a whole day and night without eating anything because there was not enough food?

☐ Never ☐ Rarely ☐ Sometimes ☐ Often

28d. In the past 4 weeks, did you worry that your household would not have enough food?

☐ Never ☐ Rarely ☐ Sometimes ☐ Often

28e. In the past 4 weeks, were you or any household member not able to eat the kinds of foods you preferred because of a lack of resources?

☐ Never ☐ Rarely ☐ Sometimes ☐ Often

28f. In the past 4 weeks, did you or any household member have to eat a limited variety of foods due to a lack of resources?

☐ Never ☐ Rarely ☐ Sometimes ☐ Often

28g. In the past 4 weeks, did you or any household member have to eat some foods that you really did not want to eat because of a lack of resources to obtain other types of food?

☐ Never ☐ Rarely ☐ Sometimes ☐ Often

28h. In the past 4 weeks, did you or any household member have to eat a smaller meal than you felt you needed because there was not enough food?

☐ Never ☐ Rarely ☐ Sometimes ☐ Often

28i. In the past 4 weeks, did you or any other household member have to eat fewer meals in a day because there was not enough food?

☐ Never ☐ Rarely ☐ Sometimes ☐ Often

29. Who provided the answers to these questions?

☐ The subject (index case)

☐ Another person on behalf of the contact, specify relationship: ____________________

CLINICAL ASSESSMENT

We have finished the questionnaire portion of this form. The nurse will now take a few measurements, including your height and weight.

30. Can I look at your arms to check for a BCG scar? *Is a BCG scar present?*

☐ 1 scar ☐ 2 scars ☐ Uncertain ☐ No scars

31. About when was your most recent BCG provided?

☐ < 1 year ago ☐ 1 - <5 years ago ☐ Uncertain

☐ 5 - 10 years ago ☐ > 10 years ago

32. Arm circumference (nearest cm)

[ ][ ] cm

33. Weight (nearest 1/10 kg)

[ ][ ][ ] . [ ] kg

☐ **Check if estimated weight *(only if unable to stand)***

34. Height [ ][ ][ ] cm **or knee height if can't stand** [ ][ ] cm

35. Random Blood Sugar

[ ][ ][ ] mg/dL ☐ Not Done

Pregnancy tests should ONLY be perfomed on female participants who are between the ages of 18-50 years.

36. Is the participant pregnant?

☐ Yes, tested positive (Go to 36a)

☐ Yes, known to be pregnant (Go to 36a)

→ 36a. Approximently how many weeks have you been pregnant? [ ][ ] weeks

☐ No

☐ Unknown

☐ Not assessed, histerectomy

☐ Not assessed, female <18 years

☐ Not assessed, other reason

☐ Not applicable, participant is male

☐ Participant declines to answer or be tested

37. *If female ask:* how many weeks ago was your last menstrual period?

[ ][ ] weeks ago ☐ Refuses to answer ☐ Histerectomy

Signature: ____________________

Initials: [ ][ ][ ]

Date Completed: [ ][ ] / [ ][ ] / [ ][ ][ ][ ]
Day Month Year

Indo-US VAP Biomarkers for Risk of Tuberculosis and for Tuberculosis Treatment Failure and Relapse

INDEX CASE FOLLOW-UP VISIT FORM

Index case ID: 1 0 2 - 0 [ ][ ][ ][ ] - A

Date of evaluation: [ ][ ] / [ ][ ] / [ ][ ][ ][ ]
Day Month Year

☐ Could not contact or evaluate, specify reason: ____________________

This visit was conducted: ☐ In person ☐ By phone ☐ Other, specify: ____________________

1. Visit type:

☐ Month 1
☐ Month 2
☐ Treatment Relapse
☐ 6-mo post-tx (M12)
☐ Month 3
☐ Month 6
☐ Treatment Completion
☐ Treatment Failure

M6 visit for MDR/XDR participants only and should not be used as an end of treatment visit

CLINIC IDENTIFICATION

☐ Not Applicable

1. Indicate the participant's DMC or TU if changed since the last visit:

Designated Microscopy Center (DMC): [ ][ ][ ]

Tuberculosis Unit (TU): [ ][ ]

TB SYMPTOMS

2. Overall, how are you feeling compared to when I saw you last?

☐ Improved, but signs/symptoms still present

☐ Improved, no signs/symptoms present

☐ Worsened

☐ No change

3. Have you had any of the following since the last study visit? *(Read options aloud)*

Cough ☐ Yes ☐ No

- Duration: [ ][ ] weeks
- Coughing up blood? ☐ Yes ☐ No

**Fever**	☐ Yes	☐ No	☐ Unknown
**Excessive night sweats**	☐ Yes	☐ No	☐ Unknown
**Unexpected weight loss**	☐ Yes	☐ No	☐ Unknown
**Loss of appetite if adult or failure to gain weight if a child**	☐ Yes	☐ No	☐ Unknown

29014

Index case ID number:

1	0	2	-	0					-	A

**Pain when taking a deep breath or coughing that you feel in your chest**	☐ Yes	☐ No	☐ Unknown
**Fatigue**	☐ Yes	☐ No	☐ Unknown
**Any other symptoms, if yes, Specify :** ____________		☐ No	☐ Unknown

4. Have you been admitted to the hospital for any reason since I last saw you?

☐ Yes, still admitted

☐ Yes, before visit

☐ No

TB TREATMENT COMPLIANCE

5. When was the last time you missed taking any of your TB medications?

☐ Within the past week ☐ 1-3 months ago

☐ 1-2 weeks ago ☐ More than 3 months ago

☐ 2-3 weeks ago ☐ Never skips medications

6. Just to confirm, how many doses of TB medication have you missed in the past 30 days? ☐☐

7. How many doses of TB medication have you missed in the past 7 days? ☐

RISK BEHAVIOURS

8a. How often do you have a drink containing alcohol?

☐ Never (skip to question 9) ☐ Monthly or less

☐ 2 to 4 times a month ☐ 2 to 3 times a week

☐ 4 or more times a week

8c. How often do you have six or more drinks on one occasion?

☐ Never ☐ Less than monthly

☐ Monthly ☐ Weekly

☐ Daily or almost daily

8b. How many drinks containing alcohol do you have on a typical day when you are drinking?
(Use the Beverage Card to show the different types and sizes of drinks)

☐ 1 or 2 ☐ 3 or 4 ☐ 5 or 6 ☐ 7, 8 or 9

☐ 10 or more

29014

Index case ID number:

1	0	2	-	0					-	A

RISK BEHAVIOURS

Now I will ask you a few questions about your smoking habits.

9. Have you changed your smoking habits in the past 6 months?

- ☐ Yes (Go to 9a)
- ☐ No (Skip to 10)

9a. Which of the following do you currently smoke:

- ☐ Beedis or other hand-rolled cigarettes
- ☐ Manufactured cigarettes
- ☐ Cigars, cheeroots, or cigarillos
- ☐ Hookah
- ☐ Other
- ☐ None, do not smoke anymore

PREGNANCY

ONLY assess pregnancy status for women ages 18-50.

10. Are you pregnant?

- ☐ Yes (Go to 10a) →
- ☐ No (Go to 11)
- ☐ Unknown (Go to 11)
- ☐ Not assessed, hysterectomy (Go to 11)
- ☐ Not applicable, participant is male (Go to 10)
- ☐ Not assessed, participant unmarried (Go to 9)
- ☐ Participant declines to answer (Go to 12)

10a. Approximately how many weeks have you been pregnant? ☐☐ weeks

11. Since the last visit, have you had any of the following pregnancy outcomes?

- ☐ Live birth (full term)
- ☐ Still birth (intrauterine fetal demise >20 weeks)
- ☐ Miscarrage (=<20 weeks)
- ☐ Early termination
- ☐ No, has not been preganant (Go to 11)
- ☐ Participant declines to answer (Go to 11)

10a. Date of outcome: ☐☐ / ☐☐ / ☐☐☐☐

29014

Index case ID number:

1	0	2	-	0				-	A

HIV TESTING

12. Did the participant have an HIV test within the protocol-specified time period?

☐ Yes

☐ No (Complete Off Study Form 99A)

☐ Not applicable

13. Height: ***(Only if <21 years of age)*** ☐☐☐ *cm*

OR

Knee height:
(Only if unable to stand) ☐☐ *cm*

☐ Not Done

13. Weight: ☐☐☐.☐

☐ Check if estimated weight
(estimate only if unable to stand)

☐ Not Done

Potts Spirometry

14. Did the participant have a spirometry test performed?

☐ Yes

☐ No (Go to 14a)

☐ Not enrolled in Potts

14a. What was the reason for no assessment?

☐ Smear+

☐ Not back to daily routine

☐ Refused due to pain of nose clip

☐ Other ______________________

Signature of Data Collector: ______________________

Date Completed: ☐☐ / ☐☐ / ☐☐☐☐
Day Month Year

Initials of Data Collector: ☐☐☐
